# Long term evaluation of the safety and efficacy of local cooling anesthesia during intravitreal injections: The COOL-2 Trial

**DOI:** 10.1371/journal.pone.0349554

**Published:** 2026-06-10

**Authors:** Arshad M. Khanani, Samir N. Patel, Charles C. Wykoff, Gun-Ho Kim, Ajay E. Kuriyan

**Affiliations:** 1 Sierra Eye Associates and University of Nevada, Reno School of Medicine, Reno, Nevada, United States of America; 2 The Retina Service of Wills Eye Hospital, Mid Atlantic Retina, Thomas Jefferson University, Philadelphia, Pennsylvania, United States of America; 3 Retina Consultants of Texas, Retina Consultants of America, Blanton Eye Institute, Houston Methodist Hospital & Weill Cornell Medical College, Houston, Texas, United States of America; 4 RecensMedical, Ulsan, Korea; 5 Department of Biomedical Engineering, Ulsan National Institute of Science and Technology, Ulsan, Korea; Sanmenxia Central Hospital, Henan University of Science and Technilogy, CHINA

## Abstract

**Background/Objective:**

To evaluate the safety and effectiveness of a medical device providing cooling anesthesia to the eye as local anesthesia for intravitreal (IVT) injections.

**Subjects/Methods:**

As a multicenter, open label, dose escalation clinical trial, subjects receiving at least 3 IVT injections in the study eye were recruited to participate. All patients received subconjunctival lidocaine as their anesthesia for previous IVT injections. Subjects receiving IVT injections were assigned (non-randomized) to four groups in which the cooling device was applied to the conjunctiva at the location of the intravitreal injection: Group 1: −10˚C for 20 seconds; Group 2: −15˚C for 10 seconds; Group 3: −15˚C for 15 seconds; and Group 4: −15˚C for 20 seconds. The primary outcome was pain during IVT injection assessed by a visual analog scale (0–10).

**Results:**

Eighty subjects were enrolled at 2 sites. The mean number of prior IVT injections was 20.3 for Group 2 and 15.0 for Group 3. Pain was significantly less for the 10 second vs the 15 second application (2.2 vs 3.6, p = 0.0255). The mean pain score was lower for Group 2 (−15°C for 10 seconds) ranging a score of 1.0 to 2.9 across the study visits, compared with Group 3 (−15°C for 15 seconds) with a mean range of 3.2 to 5.2 across the study visits. Treatment order (visit) significantly decreased by 0.10 pain units per visit (p = 0.0002). Punctate epithelial erosions (PEE) were reported in 20 of 56 eyes (35.7%) in Group 2, none were reported in Group 3.

**Conclusion:**

Cooling anesthesia device reduced the severity of pain associated with IVT injection during the procedure, although mild and transient PEE may be associated with treatment.

**Trial registration:**

ClinicalTrials.gov identifier, NCT03956797.

## Introduction

Intravitreal (IVT) injections are the most common office procedure performed by retina specialists, with over 6 million injections estimated to be performed in the United States annually [1]. This number continues to grow each year, as new therapeutics are developed for expanding indications. Major effort has been focused on making the workflow for performing IVT injections as efficient as possible as well as improving the patient experience with IVT injections.

Although these injections improve vision, patients can have associated anxiety and discomfort while undergoing this procedure. Indeed, in a survey of patients undergoing IVT injections, the step most associated with significant discomfort was the injection itself, versus the preparation or waiting, suggesting that improved anesthesia will improve the patient experience for IVT injection [2]. In another study, needle penetration was found to be one of the highest points of concern for patients during intravitreal injection [3]. Current methods of anesthesia for IVT injections include topical anesthetic drops, application of a pledget soaked with lidocaine, topical lidocaine gel, as well as subconjunctival lidocaine injection. All of these methods have respective benefits as well as tradeoffs, in terms of patient comfort as well as time of onset of anesthesia with no consensus choice for anesthetic for use in IVT injections. This is reflected in the 2019 ASRS PAT survey which found that 23% of retina specialists used topical drops, 18% used pledgets soaked with lidocaine, 25% used lidocaine gel, and 34% used subconjunctival lidocaine injection [4]. These trends are similar to other surveys of IVT anesthesia preference by retina specialists [5]. Prospective studies comparing the efficacy between different methods of anesthesia have been mixed, with one study suggesting subconjunctival lidocaine is more efficacious than lidocaine gel or topical anesthesia, while others suggest there is no difference in pain score between these three methods of anesthesia [6–9]. Systematic reviews of intravitreal injection anesthesia also have not demonstrated superiority of one type of anesthesia compared to another in pain scores [10,11]. Considerations for anesthesia for retina specialists include efficiency of the procedure, comfort of the patients, and utilization of resources and costs of these approaches. An alternative method of anesthesia that is fast and tolerable to patients with minimal adverse events may help improve both patient experience as well as workflow of retina specialists.

Cooling anesthesia is a form of nonpharmacologic anesthesia which has shown promising results in clinical studies [12,13]. We define cooling anesthesia as the local application of temperatures below freezing (usually between −10 and −20 degrees Celsius) as an anesthetic agent. This temperature is much warmer than temperatures that have been shown to cause tissue damage to the eye [14–16]. The concept of utilizing cold temperature to anesthetize human tissue is not novel, and has been used for anesthesia for injection of dermal fillers [17,18]. Mechanisms for anesthesia using cold temperature include decreased nerve conduction, which inhibits the firing of pain receptors and release of endorphins [18,19]. In addition, recent studies have suggested that cooling the surface of the eye, in lieu of pharmacologic agents, might provide effective anesthesia for IVT injections. A case report demonstrated the use of gloved ice in a glove applied to the conjunctiva and sclera of the eye for 2 minutes was sufficient to effectively anesthetize the eye for a patient with a lidocaine allergy [8]. In addition, a clinical study with a prototype cooling device demonstrated that cooling anesthesia was well tolerated and pain from cooling anesthesia was not significantly different than lidocaine gel by a visual analog scale (VAS) [20]. A first in human proof of concept clinical trial with a clinical grade device also demonstrated safety and effectiveness [12]. Here we present the results of a second clinical trial using the same clinical grade device, focused on evaluating the long term safety with repeated exposures and effectiveness of cooling anesthesia at various temperatures and durations.

## Materials and methods

This multicenter, open label, dose escalation clinical trial (ClinicalTrials.gov identifier, NCT03956797) was conducted at 2 sites (Retinal Consultants of Texas, Houston, TX and Sierra Eye Associates Reno, Nevada) in accordance with the tenets of the Declaration of Helsinki and conformed to the Health Insurance Portability and Accountability Act. The protocol was approved by Western Institutional Review Board. Participants provided written informed consent and were not compensated for participation. The study began enrolling on April 17, 2019 and completed enrollment in February 10, 2020. The last study visit was in April 2021. The authors confirm that all ongoing and related trials for this drug/intervention are registered.

### Study population

Eligible patients were 18 years of age or older who were undergoing intravitreal injection with ranibizumab (Genentech, South San Francisco, CA) or aflibercept (Regeneron, Tarrytown, NY). Subjects must have received a minimum of 3 intravitreal injections in the study eye prior to the study visit without more than mild adverse effects. Only one eye could serve as the study eye, even if both eyes were eligible.

Key exclusion criteria included the presence of scleromalacia or preexisting conjunctival, episcleral or scleral defects. Patients also could not have active severe dry eye disease not controlled with artificial tears, history of endophthalmitis with intravitreal injection or uveitis, history of retinal detachment in either eye or any history of vitrectomy. In addition, patients who received cooling anesthesia as part of the COOL-1 study (NCT NCT03732287) were eligible to enroll in the study.

### Investigational device

The cooling anesthesia device is a hand-held device that provides rapid, precise, and controlled cooling to the conjunctiva as previously described [12]. It is intended to anesthetize at the site of contact via a controlled cooling process. The device consists of three components: a hand-held device, single-use tips, and a battery charger. The single-use-tip is sterile and disposable, and the only component intended for contact with the ocular surface for a limited time (10 or 20 seconds) through a thermoelectric module within the device.

### Study design

**Fig 1:**
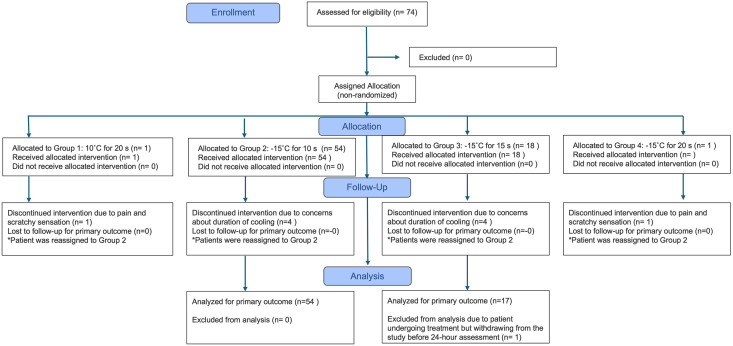
Flow diagram of the progress through the COOL-2 study.

**Fig 2:**
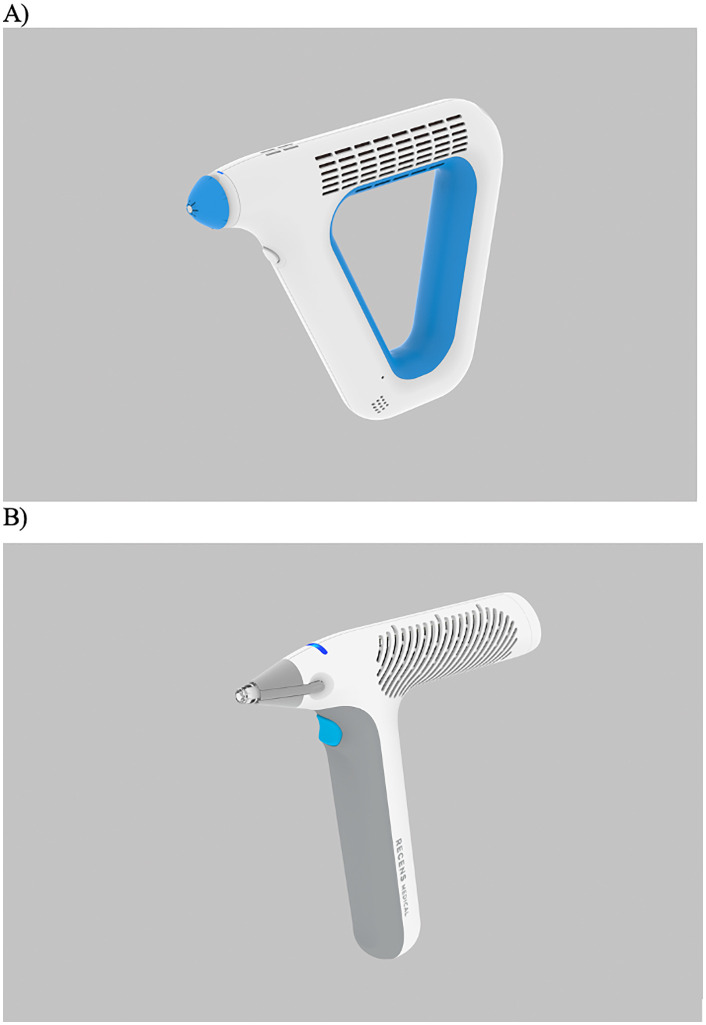
Representative images of the cooling anesthesia device. The device consists of three components: a hand-held device, single-use tips, and a battery charger. **A)** Image of initial device. **B.** Image of modified device that was introduced on October 2020. Republished from Recens Medical under a CC BY license, with permission from Recens Medical, original copyright 2020.

This was a multicenter, open-label, long-term study where subjects receiving IVT injections in the study eye were planned to be assigned (non-randomized) to 1 of 4 groups of cooling doses (i.e., temperature and duration of application) at the investigator’s discretion (**Fig 1**). The four cooling doses were the following: Group 1: Cooling device will be applied to conjunctiva at a setting of −10˚C for 20 seconds; Group 2: Cooling device will be applied to conjunctiva at a setting of −15˚C for 10 seconds; Group 3: Cooling device will be applied to conjunctiva at a setting of −15˚C for 15 seconds; and Group 4: Cooling device will be applied to conjunctiva at a setting of −15˚C for 20 seconds. Reassignment of treatment was allowed based on the clinical judgement of the investigator. A modified version of the device was introduced during the COOL-2 study in October 2020 (**Fig 2**). Compared to the original device, the modified version has various safety features such as sensors auto-detecting ocular contact and multiple timers preventing excessive cooling. It also has a reduced size and weight of approximately 40%.

Per the protocol, “the purpose of this study is primarily for safety and the study is not powered to find differences between groups in pain.” Given that, a prospective power calculation was not performed to determine the sample size needed to achieve a given power to show a difference in VAS across the four groups. However, a simulation-based estimate reflecting conditional detection probability given the observed effect size and fitted variance structure, rather than post-hoc power derived directly from the p-value can be estimated.

Focusing on groups 2 and 3, the two groups fully populated, the N for Group 2 was 54 and for Group 3 was 18. A simulation-based sensitivity analysis was conducted to assess the probability of detecting a treatment effect under the fitted mixed-effects model. The simulation preserved the observed study design, including the number of subjects, the observed number and timing of repeated measurements per subject, and the fixed- and random-effects structure of the primary analysis model (random subject intercept with AR [1] within-subject correlation). Outcome data were repeatedly resampled, assuming that the observed treatment difference (−1.36 VAS units) represented the true underlying effect, and the mixed-effects model was refit to each simulated dataset. Under these assumptions, the null hypothesis of no treatment difference was rejected at a two-sided α = 0.05 in approximately 62% of simulated trials, reflecting the conditional probability of detecting a statistically significant treatment effect given the observed effect size and variance structure.

A total of 120 subjects were planned (30 in each group) for enrollment into the study. Subjects in the study were to receive IVT injections as part of their standard treatment; the cooling treatment was planned for administration at up to 12 visits for each subject. The cooling treatment with the study device was applied at the clinic according to the subject’s assigned dose, over the site of injection prior to the subject’s planned intravitreal injection procedure. The IVT treatment was completed as per the standard of care.

The primary endpoint was pain during intravitreal injection. Pain during injection was assessed by a VAS immediately after injection. The VAS is a validated pain scale from 0 to 10 (0 = no pain, 10 = worst pain possible) and has been utilized in numerous studies studying pain after intravitreal injection. [12,21–24] This was done after the initial injection and all follow-up injections. Subject preference for anesthesia method was accessed following the first and last injections, within 5 minutes after the IVT injection and/or during a follow-up phone call subsequent to each treatment visit. Subjects were asked to indicate their preference of numbing procedure between the Cooling device, the previous method of numbing, or either method. Safety of the device after repeated applications was assessed using slit lamp exam.

### Statistical analysis

All data were analyzed using SAS Version 9.4 (SAS Institute, Inc., Cary, North Carolina, USA). Statistical analyses were pre-specified, and a *P* value < 0.05 was considered statistically significant. A repeated-measures mixed effects model was run on the pain scores taken from the 5 minute post IVT question over successive visits. The model included fixed effects for treatment duration (10 vs 15 seconds), investigator, and visit order [1–12]. Subject was a random effect and the covariance of the repeated pain scores was modeled as first order autoregressive.

The primary analysis was based on testing if pain scores differed between experimental groups. Several other tests were performed, such as at each visit and between application durations. The p-values for these tests were not adjusted for multiplicity and must be viewed as nominal only. Their purpose was not to test a hypothesis, but to explore possible determinants of pain reported.

Unpaired t-tests were used to analyze potential differences in reported pain through VAS scores between different experimental groups at each visit. As with the repeated-measures mixed effects model, only reported data were used, with no imputation of missing data. The pattern of missing data was evaluated descriptively across subjects and treatment groups (S1 Fig). Separate t-tests were run at each visit and the p-values, which are exploratory and unadjusted for multiplicity, are provided in S1 Table. Subject preference was different for the two treatment durations at the first and last injection. Differences were tested by Fisher’s exact test (see **Table 2**). Differences in pain scores between the initial and modified device were also analyzed using a mixed-effects model with adjustment for visit order and investigator. A first-order autoregressive covariance structure was used.

## Results

### Subject disposition

The seventy-four subjects analyzed were enrolled into the study at 2 investigative sites in the US, of whom 54 were assigned to Group 2, and 18 were assigned to Group 3. In the initial phase of the research due to a patient assigned to Groups 1 and a patient assigned to Group 4 experiencing a scratchy sensation and pain, the recruitment of participants for Group 1 and Group 4 was discontinued, as these groups were not incorporated into the study. Those 2 patients were reassigned to Group 2. Four other patients who were initially assigned to Group 3, were reassigned to Group 2 after 2–3 treatments in their initial group. These patients expressed concerns about the duration of cooling in their originally assigned group. All subjects completed the study except for one who received the treatment procedure at the screening visit but discontinued within 24 hours of follow up.

Baseline demographics are listed in **Table 1**. Subjects in the study had an age range of 34–94 years of age with a mean (SD) age of 71.1 (11.8) years and 79.7 (8.9) years in the 2 study groups; the majority identified as White and Not Hispanic or Latino. Of note, patients in Group 2 were significantly younger (mean age: 71.1, SD: 11.8) than patients in Group 1 (mean age: 79.7, SD: 8.9, p = 0.006). There were no significant differences in the diagnoses for treatment or the prior drug injected between the groups. The mean (SD) number of prior IVT injections was 20.3 (15.4) for Group 2 eyes and 15.0 (13.3) for Group 3 eyes (t-test, p = 0.1943). All patients received subconjunctival lidocaine as their anesthesia for previous IVT injections. Fourteen of 74 treated subjects (18.9%) had previously participated in the COOL-1 study using a similar cooling device.

**Table 1:** Baseline demographics for enrolled and treated subjects.

		Cooled at −15°C for 10 S (Group 2)	Cooled at −15°C for 15 S (Group 3)	P value^1^
Gender	Male	23/56 (41.1%)	5/18 (27.8%)	0.4065
	Female	33/56 (58.9%)	13/18 (72.2%)	
Race	White	45/56 (80.4%)	18/18 (100.0%)	0.5958
	Black	3/56 (5.4%)	0/18 (0.0%)	
	Asian	3/56 (5.4%)	0/18 (0.0%)	
	Native American	1/56 (1.8%)	0/18 (0.0%)	
	Not Reported	2/56 (3.6%)	0/18 (0.0%)	
Ethnicity	Not Hispanic or Latino	51/56 (91.1%)	17/18 (94.4%)	1.0000
	Hispanic or Latino	3/56 (5.4%)	1/18 (5.6%)	
Age (years)	N	56	18	0.0060
	Mean (SD)	71.1 (11.8)	79.7 (8.9)	
	Median	71.5	81.5	
	Q1, Q3	64.0, 80.5	73.0, 87.0	
	Min, Max	34, 94	60, 93	
Diagnosis	DME	19/56 (33.9%)	1/18 (5.6%)	0.0449
	DR	26/56 (46.4%)	14/18 (77.8%)	
	RVO	10/56 (17.9%)	3/18 (16.7%)	
	RVO/CME	1/56 (1.8%)	0/18 (0.0%)	
Prior Drug Injected	Ranibizumab	15/56 (26.8%)	11/18 (61.1%)	0.4776
	Aflibercept	8/56 (14.3%)	3/18 (16.7%)	
	Not Reported	33/56 (58.9%)	4/18 (22.2%)	
Number of Prior Injections	N	56	18	0.1943
	Mean (SD)	20.3 (15.4)	15.0 (13.3)	
	Median	17.5	11.0	
	Q1, Q3	9.0, 24.0	5.0, 17.0	
	Min, Max	3, 79	2, 48	

N = number; SD = standard deviations; Q1 = quartile 1; Q3 = quartile 3; min = minimum; max = maximum; DME = diabetic macular edema; DR = diabetic retinopathy; RVO = retinal vein occlusion; CME = cystoid macular edema.

^1^ P value using unpaired t-tests for continuous variables and Fisher’s exact test for categorial variables.

### Severity of pain

The mean pain score was lower for Group 2 (−15°C for 10 seconds) ranging a score of 1.0 to 2.9 across the study visits, compared with Group 3 (−15°C for 15 seconds) with a mean range of 3.2 to 5.2 across the study visits (**S1 Table**).

Pain was significantly less for the 10 second vs the 15 second application (estimated marginal means, 2.2 [95% CI 1.65–2.74] vs 3.6 [95% CI 2.51–4.60], p = 0.0255, respectively). Investigator was significantly different, with higher pain scores for one of the investigators (3.6 vs 2.1, p = 0.0032). Treatment order (visit) significantly decreased by 0.10 pain units per visit (p = 0.0002).

The interaction between investigator and order was explored to determine if the decrease in pain over time differed by investigator; it did not (p = 0.2439). Similarly, the interaction between treatment duration and order was explored to determine if the decrease in pain over time differed by duration. Here we did see that the shorter treatment did result in a steeper reduction in pain than for the longer treatment (−0.14 by visit, p = 0.0261). To clarify the interaction, separate models were run by treatment duration. For the shorter duration treatment (10 seconds), pain decreased by −0.13 per visit (p < 0.0001), but for the longer treatment (15 seconds), no decrease was seen (+0.02 per visit, p = .8321). Failure to show a decrease in pain for the longer duration treatment may be a function of reduced power, due to is smaller N of 17.

Of note, there was a difference in the mean pain scores with the old device (mean: 2.54, SD: 2.644) and the new device (mean: 1.48, SD: 1.949, p = 0.0281). However, as the new device was used towards the end of the follow-up, a confounder may have been introduced; pain scores reduced over time by approximately 0.10 per visit. When a mixed effects model, including investigator, treatment, and time was run, the difference between new and old device was not significant (p = 0.7446) Mean adjusted pain for old was 2.89 (SE = 0.302) and for new was 2.79 (SE = 0.392).

### Subject preference

Subject preference for anesthesia method was accessed following the first and last injections, within 5 minutes after the IVT injection and/or during the follow-up phone calls subsequent to each treatment visit. Subjects were asked to indicate their preference for a numbing procedure between the cooling device, the previous method of numbing (specify), or either method.

More subjects in Group 2 had a preference for the cooling device following the first (44/54, 81.5%; [95% CI 69.2–89.6%]) and last (35/48, 72.9% [95% CI 59.0–83.4%]) injections, compared with Group 3 form whom subject preference favored the subconjunctival method and 41.2% (7/17); [95% CI 21.6–64.0%] and 18.2% (2/11); [95% CI 5.1–47.7%] preferred the cooling device after the first (p = 0.0008) and last injections (p = 0.0020), respectively (Table 2). Of note, among the 6 patients who were reassigned to Group 2, 5 indicated they preferred cooling anesthesia over their prior anesthesia and 1 did not respond to the preference questionnaire.

**Table 2:** Anesthesia preference for enrolled and treated subjects.

		Cooled at −15°C for 10 seconds (Group 2)	Cooled at −15°C for 15 seconds (Group 3)	P value^2^
After First Injection	Cooling	44/54 (81.5%)	7/17 (41.2%)	0.0008
	Either	5/54 (9.3%)	1/17 (5.9%)	
	Subconjunctival	5/54 (9.3%)	9/17 (52.9%)	
	Not Reported	2	1	
After Last Injection	Cooling	35/48 (72.9%)	2/11 (18.2%)	0.0020
	Either	2/48 (4.2%)	2/11 (18.2%)	
	Subconjunctival	11/48 (22.9%)	7/11 (63.6%)	
	Not Reported	8	7	

^2^P value by Fisher’s exact test.

### Adverse events

Ocular adverse events (AEs) are reported in **Table 3**. Punctate epithelial erosions (PEE) were reported in 20 of 56 eyes (35.7%) (95% CI 24.8–48.8) in Group 2, 0 (95% CI 0–17.6%) were reported in Group 3. All cases were assessed by the investigator as not related to the study device; the majority were mild and resolved without sequelae. Among the 6 ongoing cases, all except 1 were mild; 1 subject experienced severe PEE on day 246 for whom several AEs of mild to moderate intensity for PEE were reported at previous visits. Other ocular AEs in Group 2 included superficial punctate keratitis (SPK) in 3 (5.4%) patients, ocular pain in 2 (3.5%) patients, ocular discharge in 1 patient (1.8%), ocular floaters in 1 (1.8%) patient, retinal detachment in 1 (1.8%) patient, and subconjunctival hemorrhage in 1 patient (1.0%). In Group 3, 1 of 18 patients experienced eyelid swelling (5.6%), which was not device-related. One patient in Group 3 experienced pain as a device-related AE (1/18, 5.6%).

**Table 3:** Incidence of adverse events for enrolled and treated subjects.

	Cooled at −15°C for 10 S (Group 2)		Cooled at −15°C for 15 S (Group 3)		P value^3^	
	**(N = 56)**		**(N = 18)**			
Ocular Adverse Events						
Punctate Epithelial Erosions	20	(35.7%)	0		0.01	
Ocular Pain	2	(3.6%)	1	(5.6%)	1.00	
Superficial Punctate Keratitis	3	(5.4%)	0		1.00	
Eyelid Swelling	0		1	(5.6%)	0.25	
Ocular Discharge	1	(1.8%)	0		1.00	
Ocular Floaters	1	(1.8%)	0		1.00	
Retinal Detachment	1	(1.8%)	0		1.00	
Subconjunctival Hemorrhage	1	(1.8%)	0		1.00	1.00
Systemic Adverse Events						
Headache	3	(5.4%)	0		1.00	1.00
Cardiopulmonary Failure	1	(1.8%)	0		1.00	
Dizziness	0		1	(5.6%)	0.25	
Nausea	0		1	(5.6%)	0.25	0.25
Peripheral Vascular Atherosclerosis	0		1	(5.6%)	0.25	0.25

^3^P value by Fisher’s exact test.

Device-related systemic AEs included dizziness and nausea in the same subject, which was ongoing at study exit and 1 case of headache, which resolved without intervention (**Table 3**). Other unrelated systemic AEs included headache, cardiopulmonary failure, peripheral vascular atherosclerosis, and death.

Assessment of ocular inflammation and the anterior and posterior segment was completed by slit-lamp examination at approximately 30 minutes after each injection. The most common slit-lamp findings were subconjunctival hemorrhage at the injection site and the corneal abnormalities related to PEE. Anterior chamber was observed as normal or deep and quiet for all subjects across all study visits. There was no evidence of persistent ocular inflammation based on slit-lamp findings.

## Discussion

Due to the continued growth in the number of intravitreal injections, significant effort has been focused on improving the patient experience with IVTs. Current methods of anesthesia for IVT injections include topical anesthesia drops (tetracaine or proparacaine), topical lidocaine gel, or subconjunctival injection of lidocaine, each of which have multiple tradeoffs for use. In this context, the current study evaluated the safety of a cold temperature novel cooling device and reports data on the first long-term study clinical trial evaluating cooling anesthesia for intravitreal injections.

Overall, the study found that the cooling device was safe and well tolerated with no serious ocular adverse events associated with the device. Although this study excluded any patient with severe dry eye requiring more than artificial tears, the use of the cooling device in the shorter duration group resulted in a higher incidence of PEE compared to the longer duration group. All cases of PEE were assessed by the investigator as not related to the study device and the majority were mild and resolved without sequelae. Furthermore, if such an AE was device-related, we would expect the longer duration group to have a higher incidence of it. This paradoxical absence of a dose-response — with the longer cooling duration (15 seconds) showing no PEE compared with 35.7% in the shorter duration (10 seconds) — is inconsistent with a direct thermal injury mechanism from the cooling device itself, as greater thermal exposure would be expected to produce more ocular surface injury. Contributing factors may include imbalance in underlying ocular surface disease between the groups (the protocol excluded only severe dry eye requiring prescription therapy, so subjects with mild dry eye using only artificial tears were eligible), the elderly population with chronic IVT exposure and ocular surface fragility, and potential mechanical factors related to device application technique. Based on these findings, the cooling device did not appear to have any long term effect on the corneal surface in a significant number of patients. Furthermore, no cases of serious ocular adverse events related to the procedure were identified including endophthalmitis, conjunctival erosion, or scleral thinning.

Multiple prior studies have used the VAS scale to evaluate pain during intravitreal injections. Current methods of anesthesia for IVT injections include topical anesthesia drops (tetracaine or proparacaine), topical lidocaine gel, as well as subconjunctival injection of lidocaine. Studies comparing the efficacy among traditional methods of anesthesia have been mixed with some suggesting subconjunctival lidocaine as more efficacious, while others suggest no difference [6,25–27]. One randomized study of 24 patients receiving four types of anesthesia during intravitreal injections (proparacaine, tetracaine, lidocaine pledgets, and subconjunctival injection of lidocaine) found no statistical difference in pain scores among the anesthetic techniques [6]. In contrast, another study of 92 patients receiving three types of anesthesia (proparacaine, proparacaine with subconjunctival injection of lidocaine, and lidocaine gel) found that subconjunctival injection of lidocaine was most effective in preventing pain during compared to proparacaine or 2% lidocaine gel [27].

Subjects in this study had an mean pain score of < 3 out 10 (maximum pain) when treated with cooling anesthesia device at −15 degrees C for 10 seconds prior to IVT injection and had a higher tendency to prefer the cooling method of anesthesia to subconjunctival injections. Subjects treated at the same temperature with higher duration had an mean pain severity of < 5, but had a lower rate of preference for the cooling method; but this result may be due to the smaller sample size treated under the condition. When using these prior clinical studies as historical controls, we found no significant difference in VAS scores of the cooling anesthesia device compared to historical controls that involved topical anesthesia methods [6,25,26]. Indeed, one prior study [6] reported mean VAS scores of 4.4 for lidocaine pledget, 3.5 for topical proparacaine, 3.8 for the subconjunctival lidocaine injection, and 4.1 for topical tetracaine, which were not significantly different to the VAS scores for Group 2 (−15°C for 10 seconds; mean, 2.2; range, 1.0 to 2.9) or Group 3 (−15°C for 15 seconds; mean, 3.6; rang, 3.2 to 5.2).

With continued use of topical anesthesia over multiple injections, there may be a concern of decreased efficacy of anesthesia on subsequent use, particularly with topical drops. For this study, we evaluated changes in VAS scores over multiple treatments and found a significant decrease in VAS with time (a 0.10 reduction per visit, p = 0.0002), suggesting a potential learning effect by investigators, patients, or both.

In terms of patient preference, over 80% of subjects reported they preferred cooling anesthesia at −15 degree Celsius for 10 seconds compared to their traditional form of anesthesia, subconjunctival lidocaine. Furthermore, patients in this study had a mean of over 15 intravitreal injections prior to enrollment and had significant experience with both forms of anesthesia. In addition to the statistical differences in the observed pain scores, the preference for cooling anesthesia underscores the clinical relevance of this alternative method for anesthesia as patients may be more adherent to treatment recommendations by minimizing patient discomfort.

This study has multiple limitations: this was an open label non-randomized clinical study without masking to the treatment effect, which may lead to selection and performance bias. Furthermore, not all study subjects were able to complete multiple study visits, which may limit the generalizability of the findings for continued effectiveness. In addition, there were baseline differences between the groups regarding mean age and diagnosis at enrollment which may confound pain perception using the cooling device. A sensitivity mixed-effects analysis incorporating baseline age as a covariate, using the same model as the primary analysis showed that age is not a significant predictor of pain and adjustment for age does not change the treatment effect. Furthermore, prior studies have reported no association between pain severity during intravitreal injection and age [28]. Additional clinical trials are warranted to evaluate the potential effectiveness of cooling anesthesia for intravitreal injections.

The results of this study indicated that the cooling anesthesia device can reduce the severity of pain associated with IVT injection during the procedure, although mild and transient PEE may be associated with the treatment. No clinically significant ocular adverse events were identified with repeated use. Furthermore, the study also demonstrated lower pain scores with more treatments, suggesting a potential learning effect. Formal comparative efficacy against standard subconjunctival lidocaine was not evaluated in this study and will be the focus of a planned future prospective, multi-center, randomized, controlled, double-masked trial.

## Supporting information

S1 Table Reported pain for enrolled and treated subjects by study visit. Data reported for subjects stratified by each study visit. Abbreviations: Q1, Q3 = First and third quartile; SD = Standard Deviation; CI = Confidence Interval.
(PDF)

S1 Fig Missingness pattern by treatment group. Within group 2 and group 3, the missingness pattern of the observed versus missing data is stratified by subject and study visit.
(PDF)

S1 File Individual data for each enrolled patient stratified by study group and visit with corresponding pain scores. (PDF)

S2 File COOL2 protocol. (PDF)

S3 File COOL2 data. (ZIP)

## Abbreviations

IVT: intravitreal
PEE: punctate epithelial erosions
VAS: visual analog scale
