# Supplementary material for: Long term evaluation of the safety and efficacy of local cooling anesthesia during intravitreal injections: The COOL-2 Trial
Source: PLoS One. 2026 Jun 10;21(6):e0349554. doi: 10.1371/journal.pone.0349554 (PMC13252741; doi:10.1371/journal.pone.0349554)
Supplement: S1 Table — Data reported for subjects stratified by each study visit. Abbreviations: Q1, Q3 = First and third quartile; SD = Standard Deviation; CI = Confidence Interval. (PDF) [file pone.0349554.s001.pdf]

		Cooled at -15°C for 10 seconds	Cooled at -15°C for 15 seconds
Visit 1	N	55	18
	Mean (SD)	2.4 (2.4)	4.2 (2.7)
	Median	2.0	4.5
	Q1, Q3	0.0, 4.0	2.0, 6.0
	Min, Max	0, 10	0, 8
	Not Reported	1	0
Visit 2	N	43	10
	Mean (SD)	2.9 (2.6)	3.2 (2.1)
	Median	2.0	3.0
	Q1, Q3	1.0, 5.0	2.0, 5.0
	Min, Max	0, 9	0, 7
	Not Reported	13	8
Visit 3	N	41	10
	Mean (SD)	2.7 (2.9)	4.2 (2.1)
	Median	2.0	4.0
	Q1, Q3	1.0, 3.0	3.0, 6.0
	Min, Max	0, 10	1, 8
	Not Reported	15	8
Visit 4	N	38	10
	Mean (SD)	2.1 (2.6)	4.3 (3.2)
	Median	1.0	3.0
	Q1, Q3	0.0, 3.0	2.0, 8.0
	Min, Max	0, 10	1, 10
	Not Reported	18	8
Visit 5	N	36	10
	Mean (SD)	1.8 (2.5)	3.3 (2.2)
	Median	1.0	3.0
	Q1, Q3	0.0, 2.5	2.0, 4.0
	Min, Max	0, 10	1, 7
	Not Reported	20	8
Visit 6	N	34	10
	Mean (SD)	1.5 (2.4)	4.2 (3.3)
	Median	0.5	3.0
	Q1, Q3	0.0, 2.0	2.0, 8.0
	Min, Max	0, 10	0, 9
	Not Reported	22	8
Visit 7	N	26	7
	Mean (SD)	1.7 (2.0)	3.4 (2.5)
	Median	2.0	3.0
	Q1, Q3	0.0, 3.0	1.0, 5.0
	Min, Max	0, 8	1, 8
	Not Reported	30	11
Visit 8	N	24	5
	Mean (SD)	1.6 (2.2)	3.8 (2.9)
	Median	0.0	4.0
	Q1, Q3	0.0, 3.0	3.0, 4.0
	Min, Max	0, 8	0, 8
	Not Reported	32	13
Visit 9	N	22	5
	Mean (SD)	1.5 (2.4)	4.0 (2.6)
	Median	0.5	3.0
	Q1, Q3	0.0, 2.0	3.0, 5.0
	Min, Max	0, 9	1, 8

	Not Reported	34	13
Visit 10	N	22	5
	Mean (SD)	1.1 (1.8)	4.8 (2.8)
	Median	0.0	5.0
	Q1, Q3	0.0, 1.0	2.0, 7.0
	Min, Max	0, 7	2, 8
	Not Reported	34	13
Visit 11	N	19	5
	Mean (SD)	1.8 (2.3)	5.2 (2.9)
	Median	1.0	5.0
	Q1, Q3	0.0, 3.0	3.0, 7.0
	Min, Max	0, 8	2, 9
	Not Reported	37	13
Visit 12	N	18	4
	Mean (SD)	1.0 (1.5)	3.5 (2.5)
	Median	0.5	3.0
	Q1, Q3	0.0, 1.0	2.0, 5.0
	Min, Max	0, 6	1, 7
	Not Reported	38	14

Supplemental Table 1. Reported Pain for Enrolled and Treated Subjects by Study Visit. Abbreviations: Q1, Q3=First and third quartile; SD=Standard Deviation; CI=Confidence Interval.
