# Supplementary figures and images for: Long term evaluation of the safety and efficacy of local cooling anesthesia during intravitreal injections: The COOL-2 Trial
Source: PLoS One. 2026 Jun 10;21(6):e0349554. doi: 10.1371/journal.pone.0349554 (PMC13252741; doi:10.1371/journal.pone.0349554)

# Missingness Pattern by Treatment Group

Group 2

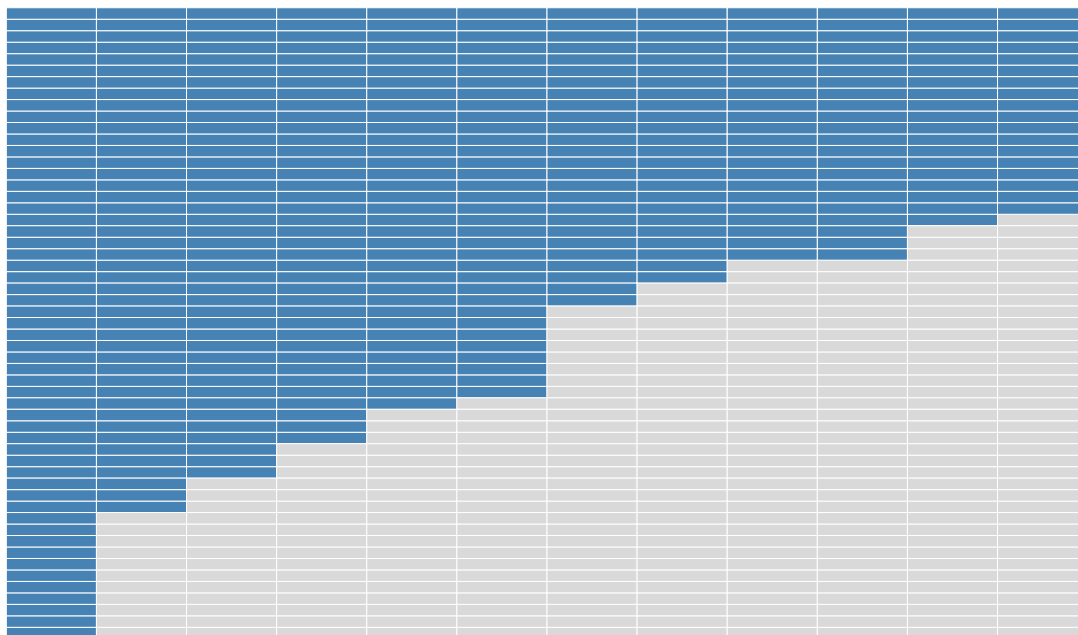

Group 3

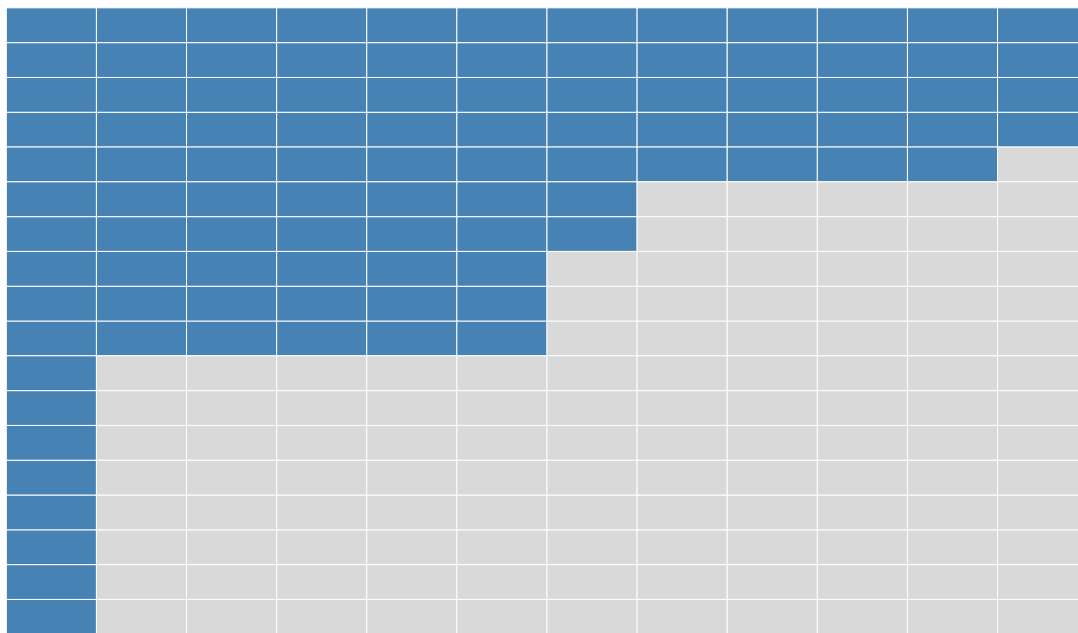

Subject

1 2 3 4 5 6 7 8 9 10 11 12

Visit (Order)

Observed Missing

Supplement: S1 Fig — Within group 2 and group 3, the missingness pattern of the observed versus missing data is stratified by subject and study visit. (PDF) [file pone.0349554.s002.pdf]
