# Supplementary material for: Long term evaluation of the safety and efficacy of local cooling anesthesia during intravitreal injections: The COOL-2 Trial
Source: PLoS One. 2026 Jun 10;21(6):e0349554. doi: 10.1371/journal.pone.0349554 (PMC13252741; doi:10.1371/journal.pone.0349554)
Supplement: S2 File — (PDF) [file pone.0349554.s004.pdf]

Long term evaluation of the safety and efficacy of Cooling anesthesia for Local anesthesia during intravitreal injection (COOL-2)

Sponsor: RecensMedical, Inc.
Room 801-8, Building 112, UNIST
50, UNIST-gil, Eonyang-eup, Ulju-gun
Ulsan, Ulsan South Korea 44919

Medical Monitor: Daniel L Chao, MD, PhD
Envision Consultants, LLC
3525 Del Mar Heights Rd Suite 732
San Diego, CA 92130

Investigators:

Arshad Khanani, MD
Director of Clinical Research
Sierra Eye Associates
950 Ryland Street Reno Nevada

Charles Wykoff, M.D., PhD
Retina Consultants of Houston, PA
6560 Fannin Street, Suite 750
Houston, TX 77030

Protocol Version 3.0

12-Feb-2020

Date Final: **Version 1.0; 12 February 2019**
Date Amended: **Version 2.0: 07 November 2019**

TABLE OF CONTENTS

I. Background and Significance ... 2
II. Criteria for Subject Selection ... 3
III. Methods and Procedures ... 4
IV. Risk/Benefit Assessment ... 8
V. Subject Identification, Recruitment and Consent/Assent ... 9
VI. Device Description ... 10
VII. Risk Analysis ... 12
VIII. Animal and Human Studies ... 13
IX. Product Development ... 18
X. Clinical Protocol Synopsis ... 19
XI. References ... 22

Purpose of the Study: The purpose of this clinical study is to evaluate the long-term safety and efficacy of cooling anesthesia application to the eye as anesthesia for intravitreal injection using a novel cooling anesthesia device.

I. Background and Significance

Retinal diseases, such as age-related macular degeneration and diabetic retinopathy are leading causes of blindness worldwide. In particular, Age related macular degeneration (AMD) is the leading cause of vision loss in individuals over the age of 60 in developed countries, and diabetic retinopathy is the leading cause of vision loss in the working class population in the United States.[1, 2] Given the growing geriatric population as well as the global epidemic of diabetes, these diseases will only continue grow in prevalence in future years.

Recently, therapeutics have been developed, which when injected into the eye, have revolutionized treatments for these diseases. In particular, antibodies against vascular endothelial growth factor (VEGF) have shown remarkable efficacy in preventing vision loss and even improving vision in both wet AMD and diabetic retinopathy.[3, 4] These therapeutics are delivered through intravitreal injection (IVT), which involves direct injection of the medication through penetration of the sclera using a small gauge (30-33 gauge) needle into the vitreous cavity. The number of IVTs performed has skyrocketed since the approval of the 1st anti-VEGF medication, Since the approval of the first anti-VEGF medication, ranibizumab in 2006, the number of IVT injections have skyrocketed, with an estimated 1 million injections per year in 2006 to around 6 million in 2016 in the United states.[6] Given the short half-life of these medications, many of these patients need to be injected at a frequency as high as once a month, creating significant treatment burden for patients as well as retina specialists. The amount of IVT injections are expected to continue to increase as new therapeutics are developed for expanding indications such as dry macular degeneration.

Due to the large volume of IVT injections in the retina clinic, significant effort has been focused on making the workflow for performing IVT injections as efficient as possible as well as improving the patient experience with IVTs. Currently retina specialists may see between 40-90 patients a day in clinic, with between a third to half of these patients requiring intravitreal injections. The ability to perform this procedure efficiently, as well as provide an excellent patient experience is critical. Despite the safety and efficacy of these IVT injections, patients can have significant anxiety and discomfort while undergoing this procedure. Indeed, in a survey of patients undergoing IVT injections, the step most associated with significant discomfort was the injection itself, versus the preparation or waiting, suggesting that improved anesthesia may improve the patient experience for IVT injection. Current methods of anesthesia for IVT injections include topical anesthesia drops (tetracaine or proparacaine), topical lidocaine gel, as well as subconjunctival injection of lidocaine. Studies comparing the efficacy between these three methods have been mixed with one study suggesting subconjunctival lidocaine is more efficacious than the other two, while others suggest there is no difference in pain score between these three methods of anesthesia.[7, 8] All of these methods have their tradeoffs include either decreased efficacy of anesthesia (topical drops), long preparation and increased expense (lidocaine gel), or extra physician time and presence of subconjunctival hemorrhage (subconjunctival lidocaine). Thus, an unmet need is an effective method of

anesthesia that is fast, tolerable to patients, and has minimal adverse events.

Other than anesthesia through pharmacologic agents, recent reports have suggested that cooling the surface of the eye may provide effective anesthesia for IVT injection. Low temperature is thought to be provide anesthesia through deceased nerve conduction as well as potentially vasoconstriction and upregulation of endorphins. A recent case report demonstrated effective anesthesia for IVT injection through placement of ice on the surface of the eye for 2 minutes in a patient with a lidocaine allergy.[9] This suggests that cooling the eye surface cooling may serve as an alternative method of anesthesia. To this end, we have a developed a novel cooling device which can rapidly cool the surface to the eye to temperatures just below freezing (-10˚C or -15˚C). We have performed animal safety studies as well as pilot human studies with a previous prototype to demonstrate safety as well as efficacy of this type of cooling anesthesia. We will now proceed with a phase I clinical trial to evaluate safety and efficacy in a clinical grade device that is eligible to be FDA approved.

II. Criteria for Subject Selection

Number of subjects: Up to 120 subjects are expected to participate at two sites.

Gender: Both male and female gender will be enrolled without any gender restrictions. Pregnant women will be excluded due to theoretical teratogenic effects of anti-VEGF medications.

Age of Subjects: Adults over the age of 18 will be enrolled in this study.

Racial and Ethnic Origin: No enrollment restrictions will be made based on racial or ethnic origin.

Inclusion Criteria:

- Men and women > 18 years old at screening visit.
- Men and women who are undergoing intravitreal injections in either one eye or both eyes with either Lucentis or Eylea regardless if they are participating in the research study or not.
- Subject has received a minimum of 3 intravitreal injections in the study eye prior to the study visit without more than mild adverse effects
- Subject is willing and able to sign the study written informed consent form (ICF).

Exclusion criteria:

- History of presence of scleromalacia
- Preexisting conjunctival, episcleral or scleral defects
- Less than 18 years of age
- Unable to provide informed consent
- Has received less than 3 injections in the study eye
- Active severe eye disease not controlled with artificial tears and requiring Restasis or Xiidra drops.
- History of Endophthalmitis with intravitreal injection
- History of uveitis

- History of retinal detachment in either eye
- History of vitrectomy

Note: subjects who received administration of cooling anesthesia as part of the COOL-1 study will not be excluded and are eligible to participate in this study.

Vulnerable Subjects: No vulnerable subjects such as children, pregnant woman, nursing home residents, students, employees, fetuses, prisoners, or persons with decisional incapacity will be recruited.

III. Methods and Procedures

Methods and Procedures:

This is multicenter, open label study (see protocol synopsis). Subjects receiving intravitreal injections, will have one eye selected as the study eye and will be assigned to a group at the discretion of the investigator. These will be one of 4 possible settings:

Groups:
Group 1: Cooling device will be applied to conjunctiva at a setting of -10˚C for 20 seconds.
Group 2: Cooling device will be applied to conjunctiva at a setting of -15˚C for 10 seconds.
Group 3: Cooling device will be applied to conjunctiva at a setting of -15˚C for 15 seconds.
Group 4: Cooling device will be applied to conjunctiva at a setting of -15˚C for 20 seconds.

Investigator can reassign subject to a different group at their discretion.

This study will be conducted within a normal intravitreal injection procedure. These **subjects will be undergoing intravitreal injection regardless of their participation in this study, and thus the intravitreal injection is not included as part of the study**. In lieu of the typical anesthesia a subject would receive for intravitreal injection (e.g. topical eye drops or subconjunctival injection of lidocaine), they would receive application of the cooling device over the site of injection for 10, 15 or 20 seconds. All of the rest of the intravitreal injection procedure will be the same as their standard visit and would occur regardless of their participating in this study.

After informed consent is obtained the following procedures will be performed:

1. Demographics Information (date of birth, gender, race & ethnicity).
2. Abbreviated history of the study eye which will include diagnosis for intravitreal injection, previous number of intravitreal (IVT) injections, previous drug used for IVT injections and previous numbing method for IVT injections.
3. SPEED Questionnaire
4. Fluorescein staining of ocular surface will be performed pre-injection by a trained investigator, to see if there is an acute effect of the cooling application. Staining will be graded on a 5-point scale:

   0) No Staining

    1) Trace
    2) Mild
    3) Moderate
    4) Severe

5. Ophthalmic Exam (slit lamp and indirect) with pupil dilation will be performed in the study eye pre-injection and 30±15 minutes post-injection. The ophthalmic exam will include assessments of the eyelids, sclera, conjunctiva, cornea, anterior chamber, iris, lens, vitreous, retina, optic nerve, vessels, peripheral retina, and absence of neovascularization. Any abnormal findings should be described.
6. Lucentis or Eylea IVT injection procedure of the study eye will be carried as defined by institutional standards.
7. Cooling anesthesia device will be placed on subject's study eye in location of investigator's discretion and will be activated, between 10 and 20 seconds as defined by the assigned study group, prior to IVT injection.
8. Injecting investigator will assess subject movement during intravitreal injection using the following criteria:
    0) No movement
    1) Mild movement
    2) Marked movement
9. 5 ± 2 minutes after IVT injection, a delegated study staff will come in and ask about
    1) pain during injection, and
    2) pain right now (5±2 minutes after injection) based on the pain visual analog scale (Figure 7).
10. Fluorescein staining of ocular surface will be performed 30 ± 10 minutes post injection by a trained investigator, to see if there is an acute effect of the cooling application. Staining will be graded on a 5-point scale:
    0) No Staining
    1) Trace
    2) Mild
    3) Moderate
    4) Severe
11. Adverse Event Review

    An adverse event (AE) is any untoward medical occurrence in a subject enrolled in the study, regardless of treatment assignment that does not necessarily have a causal relationship with the study medical device. An AE can therefore be any unintended disease or injury, or on toward clinical signs in subjects, whether or not related to the investigational medical device.

    AEs to be captured include those identified during the ocular exam, post injection pain assessment or follow up phone calls.

    The severity of an AE will be graded by the investigator using a 5-point scale and reported in detail as indicated in eSource and/or SAE form, as appropriate.

Table 1: AE Intensity Grading

Grade	Description
Grade 1	Mild; asymptomatic or mild symptoms; clinical or diagnostic observations only; intervention not indicated.
Grade 2	Moderate; minimal, local or noninvasive intervention indicated; limiting age appropriate instrumental activities of daily living (ADL).
Grade 3	Severe or medically significant but not immediately life-threatening; hospitalization or prolongation of hospitalization indicated; disabling; limiting self-care ADL.
Grade 4	Life-threatening consequences; urgent intervention indicated.
Grade 5	Death related to AE

Table 1, AE Intensity Grading

The Investigator or its designee will probe, via discussion with the subject, for the occurrence of AEs during each subject visit and record the information in the site's source documents. Adverse events will be recorded in the subject source. Adverse events will be described by duration (start and stop dates and times), intensity, outcome, treatment and relation to study medical device, or if unrelated, the cause. Subconjunctival Hemorrhages will not be considered an Adverse Event.

The causality of each AE must be assessed by an Investigator.

A suspected adverse event is any event for which there is a reasonable possibility that the medical device caused the adverse event. "Reasonable possibility" means there is evidence to suggest a causal relationship between the medical device and the adverse event.

The relationship to the intravitreal injection procedure or to the medical device will be assessed using the following definitions:

- Unrelated: There is no valid reason for suspecting a possible case-and-effect relationship between the investigational medical device or the injection procedure and the occurrence of the AE
- Related: If there is any valid reason, even if undetermined, for suspecting a possible case-and-effect relationship between the investigational medical device or the injection procedure and the occurrence of the AE.

A SAE is defined as any AE occurring during any study phase and at any dose of the investigational product or comparator, that results in any of the following outcomes:

- Results in death
- Is life-threatening adverse experience
- Requires inpatient hospitalization or prolongation of existing hospitalization
- Persistent or significant disability/incapacity
- A congenital anomaly/birth defect
- An important medical event

Other important medical events may also be considered an SAE when, based on appropriate medical judgment, they jeopardize the subject or require intervention to prevent one of the outcomes listed.

All SAEs that occur after any subject has been enrolled, before treatment, during treatment, or during the study participation, whether or not they are related to the study, must be recorded on the forms

12. A 1-2 business day post injection phone call will be performed to assess pain by delegated study staff, using the same visual analog pain scale as mentioned previously.

13. Steps 3-12 as noted above will be repeated at each clinic visit when the subject will be receiving an intravitreal injection in the study eye. Cooling anesthesia will be applied no sooner than three weeks between administration. Patient will receive up to 12 administrations of the cooling anesthesia.

14. Patient Preference Information (PPI) is accessed as below.
    A phone call after finishing the subject's last visit will be performed to access overall PPI using the question, "If you were to choose the numbing procedure, would you prefer the cooling device or the previous method of numbing (specify) or either method?
    □ previous numbing (topical or subconjunctival), □ cooling device, □ either"

    Additionally, optional PPI can be implemented as below.
    a. 5 minute post injection question, "If you were to choose the numbing procedure performed before your next eye injection would you prefer the cooling device or the previous method of numbing your eye (specify)?
       □ previous numbing (topical or subconjunctival), □ cooling device, □ either"
    b. 1-2 business day phone call, using the question, "If you were to choose the numbing procedure performed before your next eye injection would you prefer the cooling device or the previous method of numbing your eye (specify)?
       □ previous numbing (topical or subconjunctival), □ cooling device, □ either"
    c. 1-2 business day phone call to access patient experience, using the questions, "How does your 24-hour post-treatment compare with SC?: □ Better, □ Worse, □ No different", "Please give your score of our product compared with SC method on a scale of 0-100", "Does the fact that cooling anesthesia does not make any cosmetic hemorrhage affect your experience with IVT?: □ Yes, □ No".

Any information captured as part of the subject's standard of care visit (such as Snellen visual acuity, intraocular pressure, etc.), occurring on the same day as the study eye's intravitreal injection will be captured as part of the subject's source.

Table 2: Schedule of Events

Activity	Visit 1	Visit 2	Visit 3	Visit 4	Visit 5	Visit 6	Visit 7	Visit 8	Visit 9	Visit 10	Visit 11	Visit 12
Informed Consent	X											
Demographics	X											
Abbreviated History	X											

SPEED Questionnaire	X	X	X	X	X	X	X	X	X	X	X	X
Fluorescein Staining (SE) (pre- IVT injection)	X	X	X	X	X	X	X	X	X	X	X	X
Pre-IVT Injection Ophthalmic Exam (Slit lamp & Indirect)	X	X	X	X	X	X	X	X	X	X	X	X
Cooling Anesthesia Administration	X	X	X	X	X	X	X	X	X	X	X	X
Pain assessment 5 ± 2 minutes post IVT injection	X	X	X	X	X	X	X	X	X	X	X	X
Fluorescein Staining (SE) 30 + 10 minutes post IVT injection	X	X	X	X	X	X	X	X	X	X	X	X
Ophthalmic Exam (Slit Lamp & Indirect) 30 ± 15 minutes post IVT injection	X	X	X	X	X	X	X	X	X	X	X	X
Review of Adverse Events	X	X	X	X	X	X	X	X	X	X	X	X
Pain assessment 1-2 business days post injection	X	X	X	X	X	X	X	X	X	X	X	X

Table 2, Visit Schedule

Data Analysis and Monitoring: Unpaired tests will be performed to analyze potential differences in reported pain between the different experimental groups, although the purpose of this study is primarily for safety and the study is not powered to find differences between groups in pain. No data safety monitoring board will be utilized. The safety of the subjects will be monitored by the PIs and they will report any serious adverse effects to the medical monitor immediately.

Medical Monitoring

The Medical Monitor, should be contacted directly at these numbers to report any serious adverse effects

Daniel Chao M.D, PhD

Data Storage and Confidentiality:

Data will be recorded on paper source documents and kept in a locked room at the clinical site. Patient information will then be coded at the clinical site and information will then be transferred to a secure online HIPAA compliant database with deidentified information by one of the clinical coordinators. Only the PI and clinical coordinators at the site as well as members of the RecensMedical team will have access to the data.

Transition from Research Participation: Patient will then resume their standard of care after the study visit.

IV. Risk/Benefit Assessment

Risk Category: The risk of this research is greater than minimal risk, as we are using an experimental cooling device as anesthesia for intravitreal injection. However, the safety of this device has been demonstrated through multiple animal as well as human studies (see animal and human studies section).

Potential Risk: Extensive animal as well as pilot human studies have been done using this cooling device **with no signs of serious adverse effects**. As the intravitreal injection will occur regardless of their participation in this study, the risks of intravitreal injection are not listed as part of this study. Common side effects of any anesthesia for intravitreal injection include the following. This would occur approximately 50% of the time.

- Bleeding on the surface of the eye (subconjunctival hemorrhage)
- Redness of the eye (conjunctival hyperemia, ocular hyperemia)
- Increased tear production
- Irritation of the eye
- Mild discomfort

Based on our extensive animal safety studies, as well as human clinical trial data with cooling devices, and a large body of literature on cryotherapy in the eye, we do not anticipate any serious vision threatening risks, transient or otherwise, with use of our device.

Protection Against Risks: Extensive animal as well as pilot human studies have been done using this cooling device with no signs of adverse effects. This particular device has been tested in a clinical trial (COOL-1) with no reported serious adverse events.

Potential Benefits to the Subject: Patient may have an improved and less painful intravitreal injection experience due to the cooling anesthesia compared to standard of care.

Alternative to Participation: The alternative to participation is standard of care anesthesia for intravitreal injection (subconjunctival injection of lidocaine, lidocaine gel, or topical anesthetic drops).

V. Subject Identification, Recruitment and Consent/Assent

Method of Subject Identification and Recruitment: Subjects will be recruited from the PI's clinical practice during normal clinic visits. Any eligible patients for the study who are coming in for their normal clinical visit will be flagged and offered recruitment into the study.

Process of Consent: Eligible subjects will be offered recruitment into the study by the study PI or one of his clinical coordinators. The nature and purpose of the study, potential risks and benefits will be discussed with the patient to indicate their interest in the study. This conversation will be done in the patient's native language, either through one of the staff who is fluent in this language or through a phone translator. Consent will be signed on paper forms which will be stored with the rest of the study materials in a locked room at the clinical site.

Subject Capacity: All subjects will have mental capacity to give informed consent.

Subject/Representative Comprehension: Every effort will be made to ensure subject comprehension about the study prior to participating. This conversation will be done in the patient's native language, either through one of the staff who is fluent in this language or through a phone translator.

Costs to the Subject: There are no costs to the patient for this study.
Payment for Participation: Patients will be compensated with a $25 stipend for participating in the study. Subject will need to finish the study and follow up phone call to receive payment. The payment is to compensate them for the extra time that is required for their visit because of the study.

VI. Device Description

The Cooling Anesthesia Device is a hand-held device that provides rapid, precise, and controlled cooling to the conjunctiva. It is intended to anesthetize via a controlled cooling process. The device consists of three components: a hand-held device, single-use tips, and a battery charger (**Figure 1**). The single-use-tip is the only part that touches a patient eye surface for limited time (10 or 20 seconds), having an area of 4×4 mm^2. A detailed description of each component is provided below. A summary of operation is shown in **Figure 2.**

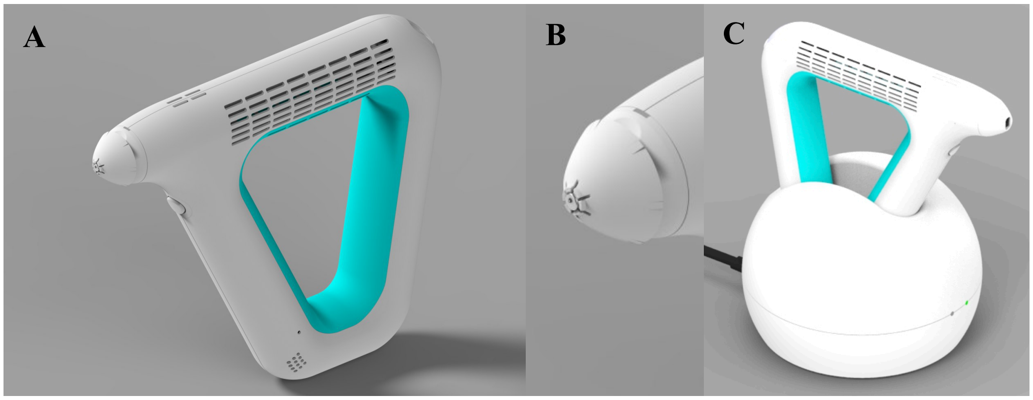

Figure 1: The components of the Cooling Anesthesia Device. (A) The hand-held device. (B) A single-use tip. (C) The battery charger.

A. Hand-held device: The hand-held device cools the single-use tips through a thermoelectric module within the device. This component also has rechargeable batteries, a display screen, and three LED indicators. The display screen shows the current temperature of the device tip. The three LED indicators (two next to the display screen and one adjacent to the single-use tip) show the cooling status of the device. Blinking blue LEDs means that the device is currently cooling. Solid blue LEDs means that the device tip has reached a preset temperature (e.g., -13˚C for -15˚C setting) and is ready for use.
B. Single-use tips: The metal surface of the single-use tips delivers the cooling treatment to the conjunctiva. This component is provided sterile and is disposed of after use.
C. Battery charger: The battery charger component is a stand that charges the batteries within the hand-held device. The device needs to be recharged about every 20 uses. The display screen shows the charging/discharging status represented in bars. When a "Low Battery" message appears on the display then the device must be recharged before it can be used.

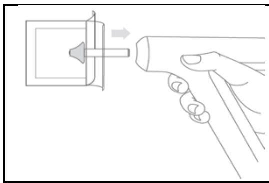	Step 1: A single-use tip is inserted into the distal end of hand-held device
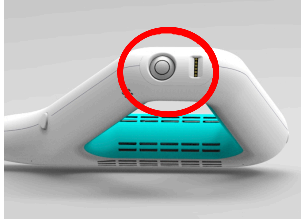	Step 2: The device is turned on by pressing the power button
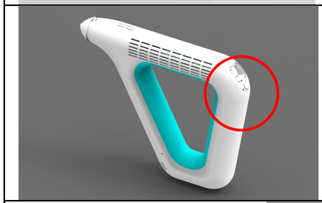	Step 3: A cooling button on the hand-held device is pressed thus initiating the cooling process
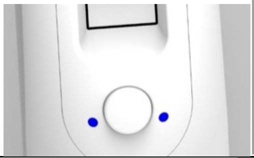 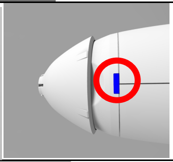	Step 4: Once pressed, the LED indicators will flash blue as the device cools. The LED indicators will be solid blue once the tip is cooled to a preset temperature and ready for use
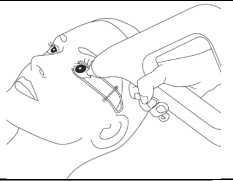 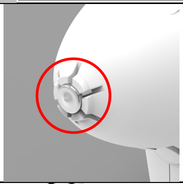	Step 5: The tip is placed on the conjunctiva
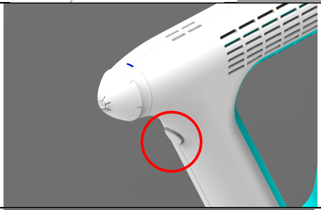	Step 6: The timer button is pressed and the temperature of the metal surface of the tip decreases to a set temperature (-10˚C or -15˚C)
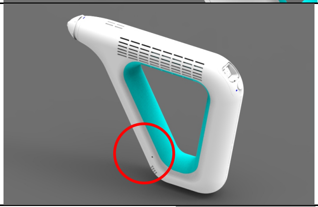	Step 7: The timer will beep once at the start, beep once at 5 seconds, and then beep twice at 10 seconds through the speaker on the hand-held device. After 10 seconds, even if the surface of the tip is against the eye, the tip temperature will increase from the set temperature (-10˚C or -15˚C) to -7˚C, at which the device makes a distinct ending beep.
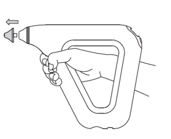 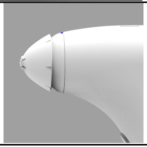	Step 8: After the distinct ending beep, the device is removed from the eye. The used tip is pulled out of the device by hand.

Figure 2: Summary of Operation for the Cooling Anesthesia Device

RecensMedical estimates that a patient will be have the Cooling Anesthesia Device applied once a month for five years. Each application lasts for 20 seconds. Therefore, per FDA's *Guidance for Industry and Food and Drug Administration Staff Use of International Standard ISO 10993-1, "Biological evaluation of medical devices - Part 1: Evaluation and testing within a risk management process"*, the Cooling Anesthesia Device is considered a surface device having

limited contact duration (≤ 24 hours) with a mucous membrane. Details about the materials and patient contact for each device component are shown in **Table 1**.

Device Component	Potential Materials	Color Additives	Contact Duration	Contact Type
Hand-held device	Thermoelectric wires, Heat-pipes, Polycarbonate FV-2 for the device enclosure	No	N/A	N/A
Single-use tips	Silver plated copper for the metal tip, Polycarbonate FV-2 for the tip cover	No	Limited	Surface device with mucosal membrane
Battery charger	Li-ion Polymer batter pack, 14.4V, 2900mAh	No	N/A	N/A

Table 1: Patient-Contacting Components and Materials

Proposed Indications for Use
RecensMedical proposes the following indication for use for the Cooling Anesthesia Device:

The Cooling Anesthesia Device is intended for topical application to the sclera, a mucous membrane. The Cooling Anesthesia Device controls pain associated with intravitreal injections.

VII. Risk Analysis:

Justification of Cooling anesthesia device as a nonsignificant (NSR) medical device

We are confident that our cooling anesthesia device is a **nonsignificant risk device** given 1) previous published studies using cryotherapy to the eye, 2) animal safety studies we have performed with a prototype device and 3) human safety studies performed with a prototype device as well as this current device. Cryotherapy is an FDA approved treatment for ocular tumors and retinal tears for over 50 years. This involves using a cryoprobe which then rapidly freezes the tissue to cause tissue damage and scarring at a temperature to -80˚C to treat ocular tumors and retinal tears. As our device is limited to a lower limit of a temperature of **-15˚C**, we are confident based on previous studies with cryotherapy on the eye in the literature, as well as our own animal safety studies that there is minimal risk to the eye. In addition, a **human clinical trial (**Clinical trials.gov (NO: NCT02872012)) performed with a prototype of our device **had no serious adverse events**, and this device was deemed a nonsignificant risk device by the University of Michigan IRB for this trial. In addition, a current human clinical trial with this device (COOL-1; NCT NCT03732287) is currently underway with no serious adverse events after 10 patients and this device was deemed a nonsignificant risk device by the Western Institutional Review Board (WIRB). **In conclusion, our extensive animal safety studies as well as human clinical data have demonstrated no serious adverse effects, and the literature**

on cryotherapy on the eye strongly supports that there would be no structural damage to the eye. Thus, we feel that our cooling anesthesia device is a nonsignificant risk device.

VIII. Animal and Human Studies

Previous studies with cryotherapy and the eye

Studies by Chi and Kelman investigated corneal endothelial viability following the application of cryotherapy at various temperatures to the cornea for 20 seconds. They observed no endothelial cell loss at -10 ˚C with largely reversible cell loss at -20˚C.[10] Only at major lower temperatures of irreversible -80˚C, with larger probe tips was their irreversible damage. Another study by Maumenee and colleagues [11] applied a cryotip at -78°C to a rabbit cornea for 5 seconds (6 mm treatment spot). They found cellular damage within the first 5 days that completely resolved at days 10 and 12. Curtin and colleagues [12] examined the effect of -40°C applied to rabbit sclera via histopathology. The temperature was applied until retinal whitening was seen (generally 10-20 seconds). The authors reported minor cell loss at days 2 and 4 post treatment. These changes were no longer apparent at days 7, 14, and 21 post treatment. In a separate study, scleral bursting strength was found to remain stable following 6 cryotherapy treatments of -60˚C applied for 1 minute each.[13] Overall, this suggests that even at temperatures that are orders of magnitude lower than the temperatures we plan to use (-10˚C , -15˚C), there is no evidence of irreversible histologic damage to the sclera or cornea. Furthermore, the lowest temperature of our device is -20˚C at its maximum performance, and any device malfunction results in a temperature warmer than -20˚C.

More recently, histology of human cadaveric eyes after cryotherapy application was studied using atomic force microscopy. A cryoprobe at -80˚C was used for treatments. Three groups were treated for 5, 10, or 20 seconds respectively (N=6 in each group). One group received a sham treatment (room temperature probe applied to sclera, N=6) and one group did not come into contact with the probe (control, N=6). All scleral sections were examined via atomic force microscopy. Lee and colleagues found that eyes treated at -80˚C for 5 seconds showed mild changes in scleral thickness and collagen fibril density (mild increase due to inflammation). These changes were more marked in the eyes treated for 10 or 20 seconds. Eyes treated for 5 seconds had no significant nanostructural differences in the diameter of collagen fibrils compared to controls. There were significant changes in eyes treated for 10 or 20 seconds. [14] This study showed that temperatures that are designed to cause tissue damage and are many times colder than our device is capable of achieving, do not cause significant damage if applied for short periods of time. Studies of cryosurgery have demonstrated reversible nervous system blockade at temperatures ranging from 0°C to -20˚C, with no pathologic tissue damage.[15] In conclusion, g**iven these previous historical studies with cryotherapy, we feel confident that cooling anesthesia at -10˚C or -15˚C for a duration of 20 seconds should not cause any clinically significant damage to the human sclera.**

Animal Safety Studies with Prototype Device

To further confirm the safety of our device, we performed non-GLP Animal safety testing was performed using a previous prototype of the current anesthesia device.[16] This device was capable of achieving temperatures as cold as -40˚C.

Methods:

Freezing treatment: To test the safety of the cooling anesthesia prototype device, we performed experiments in New Zealand rabbits, a common animal model to demonstrate safety for ophthalmic devices. New Zealand rabbits received freezing treatments just posterior to the limbus at the 1:30, 4:30, 7:30, and 10:30 meridians in both eyes (Figure X). Treatment was performed at the following temperatures (20˚C, 0˚C, -5˚C, -10˚C, -15˚C, -20˚C, -25˚C, -30˚C, -35˚C and -40˚C) for either 30 seconds duration (Phase 1; n=10 rabbits) or 10 second duration (Phase 2 n=10 rabbits).

Histology**:** Rabbits were then sacrificed 1 week after application of the cooling anesthesia device. Eyes were enucleated and sent to Comparative Biosciences Inc. for processing and histopathologic evaluation. Histopathology was graded on an industry standard 1-4 scale (Table 1). Comparative Biosciences, Inc. offers GLP and non-GLP preclinical toxicology, efficacy, pharmacology, pharmacokinetics-pharmacodynamics, histopathology, and safety studies on all laboratory species. They are a fully-staffed, state-of-the-art, AAALAC-accredited purpose-built facility with both an in-house histopathology laboratory and a full-time quality assurance unit. They are registered with the FDA, USDA, and OLAW. Histopathology was read by certified veterinary pathologist.

Animal Safety Results

None of the eyes manifested signs of clinical toxicity immediately following treatment. Histopathologic toxicity at the limbal zone was minimal and consisted of small microhemorrhage, with scattered lymphocytes and monocytes and some local edema seen in a few eyes. Interestingly, the majority of these findings were located in myocytes (ocular muscle cells) However, no ocular muscles were treated in any animal during this study. This suggests that most of the histopathology findings are likely related to manipulation of tissues and ocular muscle using the 0.5 forceps to position the rabbit eyes for cooling anesthesia administration (Figure 3B).

Animal Testing Summary

In conclusion, in all rabbit eyes treated in this study, there was no histopathologic damage noted in either the anterior segment **(cornea, iris, ciliary process, lens, sclera) as well as the posterior segment (the retina and optic nerve). None of the eyes, even those treated at the lowest temperature (-40 ˚C for 30 seconds) demonstrated any histopathologic findings related to the freezing treatment.** Given that our coldest temperature to use in humans is -15 ˚C (approximately 3 times less than the temperature used in rabbit studies), we feel confident that this will not cause any damage to the human eye.

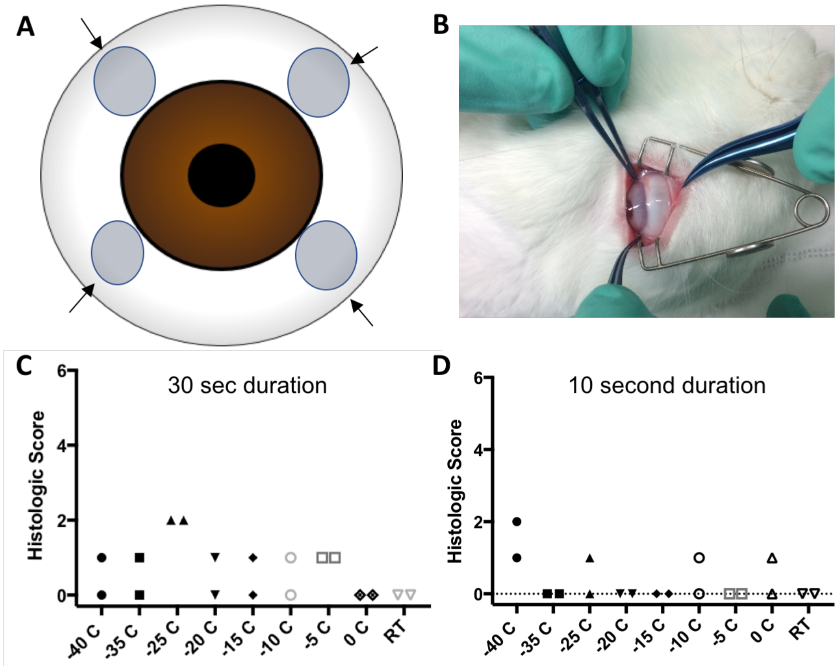

Figure 3. Animal Safety Studies with Prototype Device. A. Schematic are quadrants where cooling anesthesia is applied at 1:30, 4:30, 7:30 and 10:30 clock hours (arrows pointing to grey circles). B. Photograph of manipulation of rabbit eye for freezing treatment. C. Histologic scores for dose ranging temperatures at 30 second duration. Note: scores of 2 were related to noted myocyte damage. As our probe was not administered on ocular muscles, this was likely secondary to iatrogenic crush damage from the forceps to rotate the eyes (picture in B). D. Histologic scores for various temperatures at 10 second duration. Note: scores of 2 are related to myocyte damage, which is unlikely to be secondary to cooing anesthesia administration.

Grading System:

0= **Normal –** Tissue Considered to be normal under the conditions of the study and considering the age, sex and strain of the animal concerned. Alterations may be present which, under other circumstances, would be considered deviations from normal.

1= **Trace** – The amount of change barely exceeds that which is considered to be within normal limits.

2= **Mild** - In general, the lesion is easily identified but of limited severity. The lesion probably does not produce any functional impairment.

3= **Moderate** – The lesion is prominent but there is significant potential for increased severity. Limited tissue or organ dysfunction is possible.

4= **Severe** – The degree is either as complete as considered possible or great enough in intensity or extent to expect significant tissue or organ dysfunction

Ongoing animal safety studies with clinical grade device shows no evidence of toxicity
As we have now produced a clinical grade device that is able to go for FDA approval, we have repeated non- GLP animal safety studies with this new device. Below is the proposed animal testing schema. This testing is performed by Daegu-Gyeungbok Medical Innovation Center, a licensed CRO in Daegu, South Korea.

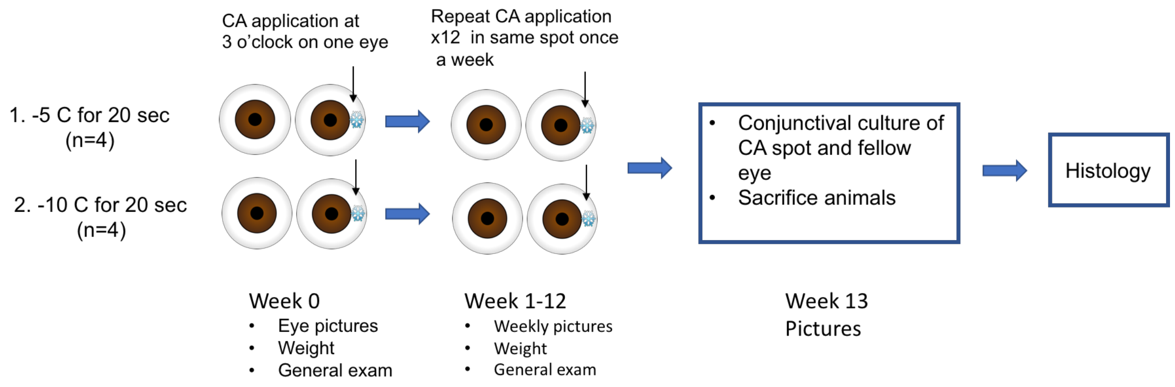

Figure 4. Ongoing animal safety experiments. 2 experimental groups will be tested, -5˚C for 20 seconds and -10˚C for 20 seconds. A single cooling anesthesia application will be applied in one eye of New Zealand White rabbits. This will be repeated 24 times, once weekly. Photos of the eye, as well as weight, and general clinical exam will be performed weekly. At week 13, a conjunctival culture will be taken at the CA spot as well as of the fellow eye of each rabbit to test for changes in the microbial flora on the conjunctiva. Animals will then be sacrificed, and histological analysis of the eyes will be performed.

Current pilot data shows that after 20 administrations of the cooling anesthesia at -5˚C or -10˚C for 20 seconds one week apart, there is no evidence of damage to the conjunctiva or adhesions based on high resolution photograph (Figure 5). We will plan to sacrifice the animals at 24 weeks, after 24 weekly administrations of cooling anesthesia to determine if there is any histologic damage (estimated end date for February 2019).

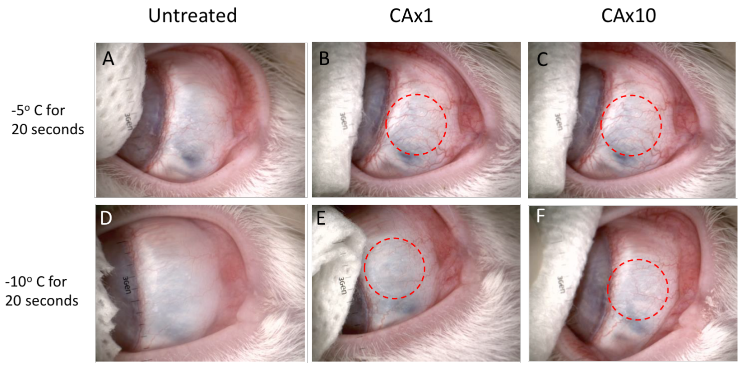

Figure 5. No evidence of conjunctival changes after 10 administrations of cooling anesthesia (CA) at either -5 of -10˚C. A-C. (A) Untreated eye prior to CA application. (B) Representative photo of rabbit eye s/p CA administration (-5˚C for 20 seconds (red circle)). Note no changes in conjunctiva. (C) Same rabbit s/p CA x10 (red circle). Note no changes in conjunctiva. (D) Untreated eye prior to cooling anesthesia (E) Representative photo of rabbit eye s/p CA administration (-10˚C for 20 seconds (red circle)) Note no change in conjunctiva. (F) Same rabbit eye as E s/p CA administration x10 Note no change in conjunctiva.

Pilot Human Clinical Studies with prototype CA device shows no significant adverse events and comparable anesthesia to standard of care

To demonstrate the safety of this device in humans, a pilot clinical study was done with a previous prototype of this device. This clinical protocol was approved by the University of Michigan Institutional Review Board, where they deemed the device a nonsignificant risk device.

A single center first in human dose ranging center was performed at the University of Michigan. (Clinical trials.gov (NO: NCT02872012)). Patients receiving bilateral intravitreal injections for either diabetic macular edema or macular degeneration were recruited into the study. One eye was randomized to receive lidocaine gel (n=22) while the other eye was randomized to receive a freezing treatment of varying temperature and duration (-5 to -10˚C for between 10-20 seconds, (n=22 total)).[5]

Safety assessment via slit lamp biomicroscopy and ophthalmologic exam as well as pain assessment through a visual analog scale was performed. **Of note there was no discomfort associated with application of the device, there were no serious adverse events, and no unexpected adverse efforts at any dose.[5] In addition, pain scores were not significantly different than the standard of care (lidocaine gel) either during injection of post injection.[5] (Figure 6).** This human study further validates that safety and efficacy of cooling anesthesia for intravitreal injection.

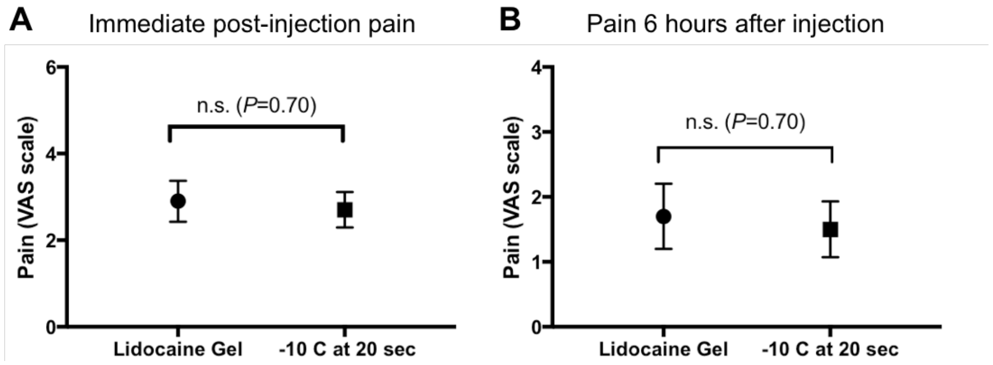

Figure 6. Cooling anesthesia clinical study results. A. Immediate post-injection pain between lidocaine gel (standard of care) group and cooling anesthesia at -10˚C at 20 seconds. No significant difference in pain scores between two groups. N=22, data represented as mean ± SEM. B. Pain 6 hours after injection between lidocaine gel (standard of care) group and cooling anesthesia at -10˚C at 20 seconds. No significant difference in pain scores between two groups. N=22 per group, data represented as mean ± SEM. Data from [5].

Figure 6: Cryoanesthesia for Intravitreal Injections (NCT02872012)

We initiated first in human clinical studies with this current clinical grade cooling device to determine safety and efficacy of cooling anesthesia for intravitreal injection. (COOL-1; NCT NCT03732287). This was a multicenter open label dose escalation study to assess safety and efficacy of the cooling device for intravitreal injection after a single administration of cooling

anesthesia. This study is currently ongoing, and 10 subjects have completed the study. There have been no adverse events and subjects tolerated anesthesia well for intravitreal injections. Given the lack of adverse events, efficacy of anesthesia, as well as no damage seen by our animal experiments after 20 administrations of cooling anesthesia, we are proceeding with a clinical trial to evaluate long term effects of repeated administrations of cooling anesthesia. Given our safety data as well as animal data as well as the literature on cryotherapy, we do not expect any serious adverse effects for this procedure.

IX. Product Development

The following testing has been conducted on the cooling anesthesia device at qualified external testing labs to adhere to international standards of the International Electrotechnical Commission (IEC) and International Organization for Standards (ISO) for medical devices suitable for human use. It has been determined that the test sample units were in compliance with the testing conducted per the requirements of the specified standards. The list of testing conducted is provided below, including the status of each test. Its full testing and certification are planned to be completed by November 2018

1. Electrical and EMC Safety

1.1. IEC60601-1 Edition 3.1
- Patient leakage current test (10uA/50uA): PASS
- Touch current test (100uA/500uA): PASS
- Dielectric strength test (1KV): PASS

1.2. IEC60601-1-2 Edition 4
- Electromagnetic Interference (Class A): PASS (5.8 dB margin)
- Electrostatic Discharge (8KV/15KV): PASS

1.3. IEC62133
- Secondary cells and batteries containing alkaline or other non-acid electrolytes: PASS (CB Certificate No. JPTUV-089703)

2. Biocompatibility

2.1. ISO10993-1 & ISO10993-5
- Biological evaluation - In-vitro Cytotoxicity: PASS (PBR Study No. GC17058)

2.2. ISO10993-1 & ISO10993-10
- Biological evaluation - Irritation: PASS (PBR Study No. GB17066)
- Biological evaluation - Skin sensitization: PASS (PBR Study No. GG17059)

3. Sterilization

3.1. ISO11137-1
- Sterilization of health care products - Radiation: PASS (Greenpia Report No. 171108-E-1)

3.2. ISO11607-1 & ISO11607-2
- Packaging for terminally sterilized medical devices: PASS (Greenpia Report No. 170911-R-1

4. Additional safety features

- At its maximum power, the device achieves a temperature of -15˚C. Any device malfunction would result in an increase in this temperature rather than a decrease

- Prior studies and our own histological studies with rabbits indicate that this minimum temperature -15˚C is not low enough to cause ocular tissue damage, even with extended exposure. For the purpose of the study, the device will not be set to a temperature colder than -15˚C.
- A temperature regulating feedback loop maintains highly accurate temperature control, and an integrated timer controls cooling time.
- The device tip is replaced with a new sterile tip before each use to help ensure safe contact with the eye surface.
- Device tip indents the eye surface, guiding subsequent needle placement.
- Ergonomic and lightweight design, facilitating safe use on or near the eye.

X. Clinical Protocol Synopsis

Protocol Title:	Long term evaluation of the safety and efficacy of Cooling anesthesia for Local anesthesia during intravitreal injection (COOL-2)
Protocol Number:	3.0
Purpose:	The purpose of this clinical study is to evaluate the safety and efficacy of repeated administrations of cooling anesthesia application to the eye as anesthesia for intravitreal injection
Primary Objective and Endpoint:	The primary objective is to determine the effect of cooling anesthesia application on pain during intravitreal injection
Key Secondary Objectives and Endpoints:	Key Secondary Objectives and Endpoints:<br>The secondary objectives of this study are<br>• Subject response to needle penetration by physician<br>• Pain assessed by visual analog scale at time of injection<br>• Pain assessed by visual analog scale 5(±2) minutes post injection<br>• Pain assessment at 1-2 business days after intravitreal injection via telephone interview<br>• Assessment of intraocular inflammation assessed by slit lamp biomicroscopy 30±15 minutes after injection<br>• Assessment of anterior segment via slit lamp biomicroscopy 30±15 minutes after injection<br>• Assessment of posterior segment via indirect ophthalmoscopy 30±15 minutes after injection
Structure	Multicenter, open label study
Duration:	Multiple visits depending on frequency of intravitreal injection in study eye (at most once every 4 weeks) with a maximum of 12 visits. A follow up phone call 1-2 business days occurs after each study visit.
Control:	None- single arm study
Study Groups;	The study will include up to 120 subjects.<br><br>Subjects receiving unilateral intravitreal injections will be assigned to a group below at the discretion of the investigator. Investigator can reassign subject to a different group at their discretion.

	1. Cooling device will be applied to conjunctiva at a setting of -10˚C for 20 seconds.<br>2. Cooling device will be applied to conjunctiva at a setting of -15˚C for 10 seconds.<br>3. Cooling device will be applied to conjunctiva at a setting of -15˚C for 15 seconds.<br>4. Cooling device will be applied to conjunctiva at a setting of -15˚C for 20 seconds.
Summary of Visit Schedule	Subjects will be consented, assigned to treatment group, and treated at each study visit. Subjects will receive a phone call 1-2 business days later after each study visit. A maximum of 6 study visits will be performed per subject.
Number of subjects:	Up to 120 subjects
Study Eye:	For subjects with unilateral injections, this will be the study eye.
Inclusion Criteria:	• Men and women > 18 years old at screening visit.<br>• Men and women who are undergoing intravitreal injections in either one eye or both eyes with either Lucentis or Eylea as part of their normal standard of care with a 30G needle.<br>• Subject has received a minimum of 3 intravitreal injections in the study eye prior to the study visit.<br>• Subject is willing and able to sign the study written informed consent form (ICF).<br>• Subjects who have previously received cooling anesthesia as part of the COOL-1 study are eligible to be a part of this study
Exclusion Criteria:	• History of presence of scleromalacia<br>• Preexisting conjunctival, episcleral or scleral defects<br>• Less than 18 years of age<br>• Unable to provide informed consent<br>• Has received less than 3 injections in the study eye<br>• Active severe eye disease not controlled with artificial tears and requiring Restasis or Xiidra drops.<br>• History of Endophthalmitis with intravitreal injection<br>• History of uveitis<br>• History of retinal detachment in either eye<br>• History of vitrectomy
Efficacy Measures	Pain on a 11-point visual pain scale (0 no pain, 10 severe pain)<br>See below.
Safety Measures	Adverse event reporting<br>1. Slit lamp biomicroscopy examination 30±15 minutes after injection<br>2. Dilated indirect ophthalmoscopy30±15 minutes after injection
Sample Size:	Up to 120, about 30 per group

Visual Analog Pain Scale used in the Study

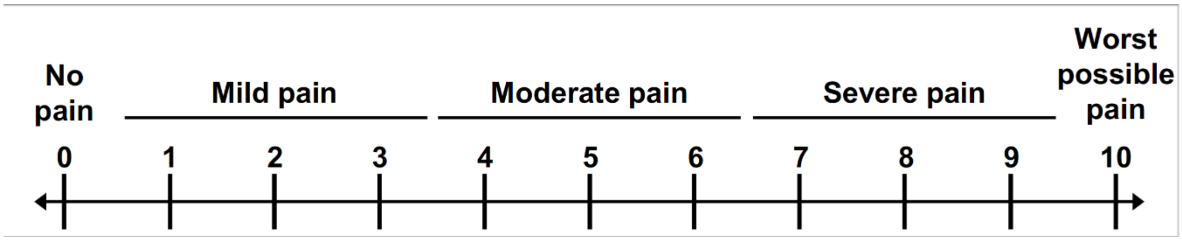

Figure 7. Visual analog pain scale used in this study. This was used in the previously presented SOLAR study (Clinical trials.gov, NCT01926977).

XI. References

1. Antonetti, D.A., Klein, R. & Gardner, T.W. Diabetic retinopathy. *N Engl J Med* **366**, 1227-1239 (2012).
2. Wong, W.L. et al. Global prevalence of age-related macular degeneration and disease burden projection for 2020 and 2040: a systematic review and meta-analysis. *Lancet Glob Health* **2**, e106-116 (2014).
3. Nguyen, Q.D. et al. Ranibizumab for diabetic macular edema: results from 2 phase III randomized trials: RISE and RIDE. *Ophthalmology* **119**, 789-801 (2012).
4. Rosenfeld, P.J. et al. Ranibizumab for neovascular age-related macular degeneration. *N Engl J Med* **355**, 1419-1431 (2006).
5. Besirli, C.S., S.; Pipe K; Kim G; Zacks, DN; Gardner TW,;Shah, A. Clinical feasibility of ultra-rapid, non-pharmacologic anesthesia for intravitreal injection in patients receiving anti-VEGF treatment. *Investigative Ophthalmology and Visual Science* **59** (2018).
6. Williams, G.A. Intravitreal injections: Health Policy Implications. *Review of Ophthalmology* (2014).
7. Blaha, G.R., Tilton, E.P., Barouch, F.C. & Marx, J.L. Randomized trial of anesthetic methods for intravitreal injections. *Retina* **31**, 535-539 (2011).
8. Yau, G.L., Jackman, C.S., Hooper, P.L. & Sheidow, T.G. Intravitreal injection anesthesia--comparison of different topical agents: a prospective randomized controlled trial. *Am J Ophthalmol* **151**, 333-337 e332 (2011).
9. Lindsell, L.B., Miller, D.M. & Brown, J.L. Use of topical ice for local anesthesia for intravitreal injections. *JAMA Ophthalmol* **132**, 1010-1011 (2014).
10. Chi, H.H. & Kelman, C.D. Histopathology of corneal endothelium after freezing. *Int Ophthalmol Clin* **7**, 369-380 (1967).
11. Maumenee, A.E. & Kornblueth, W. Regeneration of the corneal stroma cells; review of literature and histologic study. *Am J Ophthalmol* **32**, 1051-1064 (1949).
12. Curtin, V.T., Fujino, T. & Norton, E.W. Comparative histopathology of cryosurgery and photocoagulation. Observations on the advantages of cryosurgery in retinal detachment operations. *Arch Ophthalmol* **75**, 674-682 (1966).
13. Beckman, H., Leff, S. & Sugar, H.S. Scleral bursting strength. Results after treatment with ruby laser, cryotherapy, or diathermy. *Arch Ophthalmol* **93**, 428 (1975).
14. Lee, S.J. et al. Short-term effect of cryotherapy on human scleral tissue by atomic force microscopy. *Scanning* **35**, 302-307 (2013).
15. Garamy, G. Engineering aspects of cryosurgical instruments employing liquid nitrogen. *Int Ophthalmol Clin* **7**, 283-308 (1967).
16. Smith, S.K., G.; Pipe, K.P.; Besirli, C.G. Preclinical safety study of ultra-rapid, non-pharmacologic anesthesia for intravitreal injections. *Investigative Ophthalmology and Visual Science* **59** (2018).
